# Amalgam—Its Use from a Practical Standpoint

**Published:** 1895-07

**Authors:** W. E. Hasley

**Affiliations:** Brooklyn, N. Y.


### AMERICAN JOURNAL

—OF—

### DENTAL SCIENCE.

VOL. XXIX. BALTIMORE, JULY, 1895. No. 3.

ARTICLE I.

### AMALGAM—ITS USE FROM A PRACTICAL STANDPOINT.

BY DR. W. E. HASLEY, BROOKLYN, N. Y.

Read before the Second District Dental Society of New York State, Feb. 11, 1895.

The writer says that dentists should know the metals and their relative proportions when combined, so that, if satisfactory and pleasing results are obtained from the use of a certain alloy, the reason may be known; and if the opposite is the case, that the fault may be corrected. The best way to prepare an amalgam mass is by combining the alloy and mercury by weight; having first experimented until the correct proportions are obtained. The ability to use zinc phosphate and contour amalgam in combination is only acquired after considerable practice and a thorough knowledge of the working qualities of each. The powder and liquid of the zinc phosphate are placed on the slab ready for mixing, an excess of the powder being assured. The alloy and mercury of the quantity desired are weighed out in such proportions as are known to make the desired mix, placed in the mortar and triturated to amalgamation, removed to the hand and kneaded into thorough homogenity, which is quickly done, as the mass should be of decided plasticity. The amalgam is retained in the hand, the warmth of which will retard setting while the cement is being mixed. The cement should be plastic enough to allow of lining the walls of the cavity with a considerable bulk. A portion of amalgam, of about equal consistency, is quickly placed in contact and interdigitated into the cement in such a way as to cause the cement to dome in the center of the crown, and also to crowd toward the outer edge of the cavity. In this way a very strong foundation is obtained, the weak walls strengthened and a good color secured. As the cement hardens the enamel edges are cleaned of it, and the building up and contouring with amalgam continued. A number of mixes are necessary, and the last additions are thoroughly wafered, which allows of perfect contour-work, enabling the cutting away of any surplus, and the easy addition where necessary.

Gutta-percha is recognized as being a perfect non-conductor, possessed of good color, easily worked, and more nearly approaching perfect compatibility between filling and tooth-bone than any other material in the whole list, and therefore ranking first as a preventive of recurring decay, but its total lack of edge strength limits its use as a filling to those cavities which are not exposed to attrition. Zinc phosphate is possessed of excellent color, does not shrink, has great adhesive power, and is comfortably worked. It is not to be used in close proximity to a vital pulp, nor is it to be relied upon as a permanent filling, as it is subject to more or less rapid disintegration when exposed to the fluids of the mouth. Amalgam possesses all the edge-strength needed, resists attrition and the oral fluids, is so readily worked as to permit of any extent of contour-work, even to the entire replacement of a lost crown on bicuspid or molar roots, and has a desirable degree of compatibility when in contact with dentine, as is evidenced by its tooth-saving quality. However, it lacks proper color, is a conductor of electricity and thermal changes, and shrinks, bulges, and spheroids in proportion to the low grade of its alloy and the excess of mercury contained.

As a typical example, a superior second bicuspid is presented which we desire to save. The patient is young and the tooth-structure soft and frail. The whole approximal walls are lost by decay, also a large portion of the grinding surface; the fissure also is decayed deeply, uniting the approximal cavities, which are partly filled by hypertrophied growth of gum-tissue. At the first sitting the cavities are syringed with warm water, and carefully packed with cotton saturated with oil of cloves or camphophenique, to crowd out intruding gum-tissues, secure slight separation, and obtund sensitive dentine. At the next sitting the bicuspids and first molar are placed under the rubber, the edges being carefully tucked under the gum with a thin scaler without the use of ligatures, and the cavities dried. The enamel edges are carefully trimmed, conserving all that will be consistent with zinc phosphate as a liner and strengthener. The decay is carefully removed from the circumference of the cavities, and, if sensitive, obtunded with oil of cloves, eugenol, or camphophenique, avoiding any of the escharotic agents. A considerable portion of softened dentine is conserved over the region of possible pulp-exposure, and carefully protected with a thin wafer of low-heat gutta-percha, which is easily placed by picking it up with a warmed instrument touched to the wet stopper of the cajuput bottle, warming slightly over a spirit lamp, placing accurately in the cavity, and pressing the edges into apposition. The oil of cajuput softens the gutta-percha just enough to cause adherence, and also acts as an obtunder.

The first demand for the salvation of such a tooth, when the cavities are prepared, is pulp-protection; and for this gutta-percha is selected without hesitation, three wafers being necessary, one in each approximal cavity and one at the bottom of the fissure.

The second need is to guard against recurrence of decay at the cervical margin, and submarine amalgam is used, being built down to a line with the gum festoon, the last portions wafered and the edges carefully trimmed wich a very thin sickle scaler. The hardening by wafering and the careful trimming and finishing of this portion of the filling at this time is a very important point, as it never afterward can be so well and easily done. The third need is the strengthening of the frail enamel-walls and the prevention of their discoloration. Zinc phosphate ranks first in these desirable attributes, and may be used in combination with the amalgam while both are plastic, or simply as a liner mixed to a plasticity which will allow of easy and thorough adaptation to the cavity-walls with a small ball burnisher, occasionally touching the instrument to an oil-pad to prevent adhesion of the cement. And now the conditions call for a material possessed of excellent edge-strength, resistive to attrition from mastication, and of good color. Contour amalgam, when properly inserted, fulfills these conditions most admirably. Amalgamated with sufficient mercury to render the mass plastic, the first piece is placed in apposition to the previously-inserted submarine amalgam in the distal cavity at the cervix, and tapped into thorough amalgamation; each succeeding piece is inserted and tapped (not burnished) into homogenity until the distal cavity is wholly filled, contoured, and extended into the fissure; the last portions are wafered, and the lost contour is carefully restored. The mesial cavity is filled in the same manner, uniting the two fillings in the fissure. The wafering must be thorough, rendering full contouring and finishing easy, therefore insuring a minimum of shrinkage and strong edges. Such a combination filling must appeal to us all as fulfilling its mission of tooth-salvation; the pulp is protected from all outside influences; the weak walls are strengthened and color is maintained; the recurrence of decay is guarded against by a coppered amalgam at the cervical wall, and the lost contour restored. The result is a tooth of good temperature, with a prospect of years of comfortable service.—*Dental Cosmos.*

---

ARTICLE II.

### BLEACHING TEETH BY ELECTRICITY.

